# Prevalence, Infectivity, and Associated Risk Factors of Hepatitis B Virus among Pregnant Women in Yirgalem Hospital, Ethiopia: Implication of Screening to Control Mother-to-Child Transmission

**DOI:** 10.1155/2018/8435910

**Published:** 2018-08-05

**Authors:** Anteneh Amsalu, Getachew Ferede, Setegn Eshetie, Agete Tadewos, Demissie Assegu

**Affiliations:** ^1^Department of Medical Microbiology, University of Gondar, Gondar, Ethiopia; ^2^Department of Medical Laboratory Sciences, Hawassa University, Hawassa, Ethiopia

## Abstract

**Background:**

Hepatitis B surface antigen (HBsAg) and hepatitis B e antigen (HBeAg) positive mother has up to 90% likelihood of mother-to-child transmission (MTCT) of hepatitis B virus (HBV) to newborns in the absence of any prophylaxis or antiviral therapy utilization. However, routine antenatal screening and intervention strategies are not yet practiced in Ethiopia. Therefore, this study was conducted to determine the prevalence, infectivity, and associated risk factors of HBV among pregnant women.

**Methods:**

A cross-sectional study was conducted from October 2015 to August 2016 in Yirgalem Hospital. A total of 475 pregnant women were recruited, and data on sociodemography and potential risk factors were collected using a structured questionnaire. In addition, blood samples were tested for HBsAg, and HBsAg positive samples were retested for HBeAg using commercially available strip test. The status of HIV was collected from the records.

**Results:**

The seroprevalence of HBsAg was 34 (7.2%), of whom 13 (38.8%) were positive for HBeAg. The prevalence of HIV infection was 10.1% (48/475). Ten out of 34 HBV positive cases (29.4%) were coinfected with HIV. The overall HBV/HIV coinfection rate was 2.1% (10/475). Women with history of multiple sexual partners and being HIV positive were significantly associated with HBsAg positivity. Among the study participants, 35.4% were aware of MTCT of HBV and only 12 (2.5%) have taken HBV vaccine.

**Conclusions:**

High prevalence of HBsAg and HBeAg as well as low awareness and practices of HBV prevention methods suggests that perinatal transmission of HBV might be the prevailing mode of HBV transmission in the study area. Thus, screening of all pregnant women, particularly those who had history of multiple sexual partners and HIV coinfection, and provision of health education about HBV prevention methods are inevitable.

## 1. Introduction

Hepatitis B virus (HBV) is thought to be the main etiological agent for chronic liver disease (CLD) worldwide. Over 2 billion people today have been infected with HBV and 350 million of them are chronically infected, with annual death of more than 1 million of HBV-related CLD [1–3]. Mother-to-child transmission (MTCT) is responsible for approximately one-half of chronic hepatitis B (CHB) infection worldwide [4]. In endemic areas, where carrier rates are greater than 5%, perinatal transmission is common, especially when HBV-infected mothers are also HBeAg positive [5, 6]. Without any prophylaxis or antiviral therapy, women who are acutely infected with HBV or are chronic carriers of HBV are likely to transmit the virus to their offspring at the time of delivery [7].

The risk of MTCT among infants born to HBV-infected mothers ranges from 10 to 40% in HBeAg negative mothers and to as high as 90% in HBeAg-positive mothers with HBV deoxyribonucleic acid (DNA) level (>200,000IU/ml, equivalent to 6 log copies/ml). The majority (> 95%) of perinatally acquired infection results in CHB infection, due to induction of an immune tolerant state of variable duration [8, 9] and has a 15 to 25 percent risk of dying in adulthood from cirrhosis or liver cancer [10]. The risk of developing CHB infection is inversely proportional to the age at time of exposure and immune status [6, 9]. In addition, concurrent viral (hepatitis A, C, or D viruses or HIV) infection, high maternal HBV viral load and activity of viral replication (determined by detection of HBeAg) in the third trimester of pregnancy, increases the risk of perinatal transmission [6, 11].

Regardless of whether they have been previously tested or vaccinated, screening of all pregnant women for HBV infection at the first prenatal visit is important in view of the morbidity and mortality of pregnant women, its effect on the pregnancy outcome, and the risk of vertical transmission from mother to child [12]. In Ethiopia, the HBsAg prevalence rate among pregnant women varies between 3% and 7.8% [13–17]. Despite its prevalence, there is a paucity of data regarding on HBeAg status among pregnant women which helps to understand the frequency of highly infective HBV carriers in the given region which in turn helps to design and implement preventive and control measures and awareness of transmission route of HBV infection. Hence, the present study was designed to determine seroprevalence, infectivity, and associated risk factor of HBV among pregnant women attending the Yirgalem Hospital.

## 2. Materials and Methods

### 2.1. Ethical Consideration

This study was reviewed and approved by the Institutional Review Board (IRB) of Hawassa University College of Medicine and Health Sciences. Then support letter was obtained from the Yirgalem Hospital administration. The purpose and importance of the study were explained to each study participant. To ensure confidentiality of participant's information, anonymous typing was applied for every study participant. Study participants were interviewed alone to keep the privacy and all participants did not pay for the test. The test results were given to the clinicians who are working on ANC clinic of the hospital and all women who tested positive for HBsAg were counseled on their status, the route of disease transmission, the need for immunization to their neonate at birth, and close-contact screening against hepatitis. Then, they were referred to internal medicine for further diagnosis and management.

### 2.2. Study Design, Area, and Period

A cross-sectional study was conducted among pregnant women attending Yirgalem Hospital ANC clinic from October 2015 to August 2016. The hospital was located 70 km far from Hawassa, the capital city of Southern Nations and Nationalities People's Region (SNNPR), and 345 km from Addis Ababa, the capital city of Ethiopia. It is the largest hospital in the region and provides medical education and training in addition to medical care. The hospital ANC clinic gives services for more than 15 pregnant women per a day and has 30 bed rooms to serve pregnant women. At the time of their first visit of the ANC clinic, pregnant women have been screened for HIV, syphilis, anemia, and proteinuria. However, women were screened for HBV only when there is any suspicion of risk.

### 2.3. Population

Sample size was estimated to be 423 using single population proportion formula, assuming 50% HBeAg prevalence (infectivity) in pregnant women, 5% precision, and 95% level of confidence. However, in attempting to enhance the statistical power of detecting the rate difference by exposure status, we investigated a total of 500 pregnant women, prospectively. 475 consecutive pregnant women attending antenatal care (ANC) clinic in Yirgalem Hospital during the study period were enrolled. Pregnant women who are healthcare workers and refused to give consent for the blood test were exempted from the study.

### 2.4. Data Collection

#### 2.4.1. Sociodemographic Data

A written informed consent was obtained after careful explanation, about the concept of the study to each pregnant woman, before their inclusion in the study. Two midwives were trained for 2 days on study procedures, facts on HBV infections and transmission, counseling, and safety issues. Data on sociodemography and potential risk factors were collected using structured questionnaires. HIV result and ART status were obtained from their medical records.

### 2.5. Specimen Collection and Handling

About 5 ml of venous blood was collected from all pregnant women in Yirgalem Hospital Laboratory. The blood was allowed to clot and serum was separated by centrifugation at room temperature at 3000 rpm, and HBsAg was performed. About 2 ml serum was stored in the freezer at −20°C and transported to Hawassa University Teaching Laboratory using a cold box for further test.

### 2.6. Laboratory Testing

All samples were screened for the presence of HBsAg using a commercial test strip (Shanghai Eugene Biotech Co., Ltd, China). The sensitivity and specificity of the HBsAg kit were 100% and 99.34%, respectively. All samples that tested positive were retested for confirmation using the same kit and there were no discordant results. All HBsAg positive samples were retested for HBeAg using a commercial test strip, the INSIGHT HBeAg test (Tulip Diagnostics (P) Ltd, India). The sensitivity and specificity of the HBeAg kit were 99% and 97%, respectively. All tests were carried out according to the manufacturer's instructions as outlined in the package inserts. In addition, HIV result was taken from the medical records, which is a routine test recommended for all pregnant women in Ethiopia and is uniformly performed using the established national rapid testing algorithm: the Kehua Bioengineering (KHB) test kit (Shanghai, China) is used as a screening test, followed by the HIV1/2 STAT-PAK assay if positive. If the STAT-PAK and KHB results are discordant, the Uni-Gold HIV test is used as a tiebreaker to determine the result.

### 2.7. Quality Assurance

The validity and completeness of the data were checked by the trained supervisor daily. The performance of the rapid HBsAg test kit was evaluated using known positive and negative controls obtained from enzyme linked immunosorbent assay (ELISA) tested blood donors and have consistent result. Sera of positive HBeAg study subjects were retested by the same method and give the same result. Furthermore, formation of colored band to the control (C) line acts as a procedural control and serves to valid the result.

### 2.8. Data Analysis

Data were coded, entered, and analyzed using SPSS version 20 (IBM Corp., Armonk, NY, USA). We described data using either proportion or mean with standard deviation (SD). Association between participant characteristics and outcome variables (HBsAg positivity) was assessed using *χ*2 test (or Fisher's exact test as appropriate) for categorical predictors. All explanatory variables with a p-value ≤ 0.05 in the bivariate analysis were included in the multivariate logistic regression model to identify variables which have been associated independently. Odds ratios (OR) with their 95% confidence intervals (CI) served to investigate the influence of various factors on the occurrence of HBV infection. A p-value of <0.05 was regarded as significant.

## 3. Results

### 3.1. Sociodemographic Characteristics

Out of 500 pregnant women approached during the study period, 25 (5.0%) were excluded because 6 refused to participate and 19 were healthcare workers. Thus, a total of 475 pregnant women aged 18–42 years were enrolled into the study. The mean (standard deviation (SD)) age of the study group was 26.5±4.6 years, and HBV infection rate increased as the age increased. Majority, 416 (87.2%), of the women had the educational status of at least elementary and 323 (68.0%) of women were urban in residence. A large proportion of the women, 428 (90.1%), were currently married, but those who were divorced or widowed were 3.8%, and 322 (67.8%) of them were multigravida. More than half of the study participants were housewives in occupation and majority of them, 209 (44.0%), were in the third trimester (Table 1).

### 3.2. HBeAg Positivity among HBsAg Positive Pregnant Women

**Figure 1:**
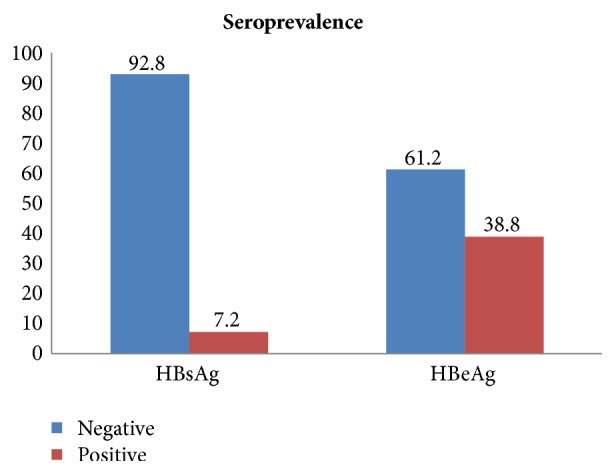
Seroprevalence rate of HBsAg among pregnant women and HBeAg positivity in Yirgalem Hospital, October 2015 to August 2016.

The overall seroprevalence of HBsAg was 34 (7.2%) (95% CI 4.9% - 9.3%). Three of them had known their HBsAg status. Among 34 HBsAg positive women, 13 (38.2%) were also positive for HBeAg (Figure 1). The highest prevalence rate of HBsAg was observed in the age group ≥ 30 years (10.5%) followed by the age group 25–29 years (6.4%); however, no statistically significant difference was observed with age groups. Almost all of the participants with HBsAg positivity were married and 70.6% were multigravida. None of the sociodemographic and obstetrical characteristics of pregnant women assessed in this study was significantly associated with HBsAg positivity (Table 1).

### 3.3. Associated Risk Factors of HBV Infection

Among 475 pregnant women, 43 (9.1%) had a history of multiple sexual partners, of which 16.3% were positive for HBsAg. Statistically significant association was detected between HBV infection and having multiple sexual partners (p= 0.02). Women having history of multiple sexual partners had higher odds of HBsAg positivity (aOR = 2.92, 95% CI = 1.19-7.16) as compared to those without history of multiple sexual partners.

In this study, 48 (10.1%) of pregnant women were HIV positive. 36 (75%) of them were on ART. Ten of 48 women were equally infected with the HBV. Eight out of 10 coinfected women started ART and six of them were HBeAg negative while two were HBeAg positive. Overall, 10 (2.1%) pregnant women were coinfected with HIV and HBV. In a bivariate analysis, those pregnant women infected with HIV were 4.4 times (aOR = 4.44, 95% CI = 1.96-10.08) more likely to be HBsAg positive than those who were HIV negative. However, place of birth, a history of previous surgery, history of blood transfusion, a history of tooth extraction or tattoos, family history of liver disease, a previous history of abortion, and being diabetes mellitus (DM) patient were not found to be significantly associated with the HBsAg status (Table 2).

In multivariate analysis of selected variables for independent predictors of HBV in pregnant women, a history of multiple sexual partners (aOR=2.92, 95%CI=1.19-7.16) and HIV positivity (aOR= 4.44, 95%CI=1.96-10.08) remained statistically significant predictors of HBV among pregnant women (Table 2).

### 3.4. Previous HBV Screening, Vaccination, and Awareness Status of Pregnant Women

Of the total participants who responded to the question whether they were previously screened or vaccinated at the time of interview, seventy-four (15.6%) women have been previously screened for HBV. Of these, 6 (8.1%) participants were currently positive for HBsAg, and six started ART but were negative for HBeAg. During oral interview, out of these six positive participants, three answered that they were positive while the other three had conducted screening and their results were negative. Out of the total participants, only 12 (2.5%) reported that they received one or more doses of hepatitis B vaccine. Of these, only 3(0.6%) pregnant women had received three doses (full dose). About 46.5%, 44.8%, and 35.4% of participants were aware of HBV transmission via sexual contact and blood and body fluids contact through nonintact skin and mucus membrane and from MTCT, respectively (Table 3).

## 4. Discussion

Early screening of pregnant women for HBV infection will have paramount importance to investigate the infection and to implement evidence based medical interventions. Since most of the pregnant women are not aware of their HBV infection status, they may serve as an important reservoir to fuel HBV transmission [18]. The seroprevalence of HBsAg in this study was 7.2%, which is in agreement with the studies in Addis Ababa, Central Ethiopia (6%)[19], Deder Hospital, Eastern Ethiopia (6.9%) [20], and Southern Ethiopia (6.1%-7.8%) [16, 17]. Similarly, the current finding was also comparable with the findings of studies in Yaounde, Cameroon (7.7%) [21], in Mali (8.0%) [22], and in Nigeria (6.67%-8.3%) [23, 24]. However, it was higher than previous studies carried out in other parts of Ethiopia (3%-4.4%) [13–15, 19, 25] and in Dares Salaam, Tanzania (3.9%) [26]. The high HBsAg positivity rate observed in this study might be due to multiple sexual practices and low level of awareness of the different routes of HBV transmission. On the other hand, the prevalence of HBsAg in this study was lower than prevalence rates of 9.7% and 10.2% reported in Cameroon [18, 27], 10.8% in Yemen [28], and 11.8% in northern Uganda[29]. These differences might be attributable to differences in the study population, whereby a selected population exposed to no condom sexual intercourse in a postconflict region with high rates of HIV infection were studied in Uganda [29], cultural practices such as circumcision in Yemen [28], and the obvious natural difference linked with various geographical situations.

The HBeAg status and the HBV viral load are both factors known to be associated with vertical HBV transmission [30]. We have assessed the presence of HBeAg which is a marker of high infectivity, as a proxy measure for the risk of vertical transmission of HBV. Out of all HBsAg positive patients, 38.2% were positive for HBeAg. This finding was significantly higher as compared to other studies elsewhere [21, 31]. This might be due to difference in diagnostic methods which used ELISA kit [21, 31]. It is known that the risk of vertical transmission and resulting chronic infection from HBsAg (+) mother to her baby is approximately 90% in HBeAg-positive pregnant women [8]. Hence our result suggests that vertical transmission is possibly an important means of HBV transmission in the study area where there is no birth dose vaccination program for newborn of HBsAg carrier mothers.

In the present study, sociodemographic variables like age, marital and educational status, residence, and occupation of participants as well as reproductive variables like gestational age and gravidity were not significantly associated with the risk of HBV infection. This finding is in line with the study conducted in Felege Hiwot Referral Hospital, Ethiopia [15] and Nigeria [32]. However, it contrasted with previous study that showed that pregnant women with no formal education had higher odds of HBV infection [16].

In the current study, pregnant women who had multiple sexual partners were almost three times more likely to have risk of acquiring HBV infection as compared to their counterparts. This is in agreement with the findings in other parts of Ethiopia [15, 20] and in Africa [31, 32]. Although 46.5% of the study participants were aware of sexual route of HBV transmission, women who have awareness had no significant difference as compared to those who have no awareness. This highlights that further health education is needed to protect pregnant women from being infected and to cut off sexual transmission of HBV through change in sexual practice and behavior modification.

In this study, high prevalence of HIV infection (10.1%) among pregnant women attending the ANC was consistent with the previous studies in Gondar (9.6-11.9%) [33–36]. HIV-infected pregnant women were more than 4 times more likely to be coinfected with HBV than HIV-uninfected ones. This is in agreement with the findings of Noubiap et al. [27] who demonstrated that HIV-infected women were 22 times more likely to be coinfected with HBV than HIV-uninfected women during pregnancy. This can be explained by the fact that HBV and HIV share common modes of transmission. Moreover, it has been reported that HIV/HBV coinfection facilitates HBV replication and reactivation, leading to higher HBV-DNA levels and reduced spontaneous clearance of the virus [37]. Although we overlooked salvaging the duration of ART taken and type of ART regimens, lamivudine is the first line ART treatment for HIV positive pregnant women in Ethiopia [38]. It has dual nucleoside reverse transcriptase inhibitor backbone in women with HIV/HBV coinfection; in line with these eight coinfected women who started ART, two women were HBeAg positive, which needs further prospective studies to investigate the effect of lamivudine in reducing HBeAg during coinfection with HIV. The overall HBV/HIV coinfection rate in our study population was 2.1%. This coinfection rate is almost three times the 0.74% rate recently reported among pregnant women in Hawassa [16], greater than the 1.3% rate reported in Northwest Ethiopia [14] and the 1.5% rate in Cameron [27], and significantly lower than the 4.2% rate reported in Nigeria [24]. The difference in coinfection rate may be due to the relatively small number of HIV positive cases in the previous study.

Since mother-child transmission is the major route of acquisition of HBV worldwide, particularly in endemic countries like Ethiopia, early recognition of HBV carrier pregnant women followed by treatment with safe antiviral agents, if indicated, and vaccination will reduce perinatal HBV infection and its complications [39]. In this study, only 15.6% of pregnant women have been previously screened for HBV. Of them, just 2.5% of pregnant women have been vaccinated against HBV. Moreover, almost two-thirds of the study participants are unaware of perinatal transmission of HBV. Thus, the provision of appropriate and correct information about the common aspects of HBV infection including perinatal transmissions, screening, and prevention by vaccination is warranted to further improve the control of HBV infection in the target group. Though there is an improvement as compared to the previous study in Hawassa [16] which reported that none of the study participants were screened and vaccinated for HBV, this difference in awareness might be because previously screened pregnant women in this study were more likely to have heard about transmission route of HBV compared to those who had no previous experience of HBV testing [40].

Nevertheless, this study has the limitation that other confirmatory methods like ELISA and molecular HBV-DNA test were not performed due to lack of budget and molecular virology laboratory facilities. The generalizability of results to all pregnant women may be limited by selection and information bias due to the institutional based nature of the study and the reliance on participants' report to assess associated factors.

## 5. Conclusions

High prevalence of HBsAg and HBeAg as well as low awareness and practices of HBV prevention methods suggests that perinatal transmission of HBV might be the prevailing mode of HBV transmission in the study area. Thus, screening of all pregnant women, particularly those who had history of multiple sexual partners and HIV coinfection, and provision of health education about HBV prevention methods are inevitable

## Figures and Tables

**Table 1:** HBsAg in relation to sociodemography in pregnant women attending Yirgalem Hospital, October 2015 to August 2016.

**Characteristics **	**Total women** **∗**	**Women with** **∗**	**COR(95**%**CI)**	**p-value **
	**(N = 475)**	**HBsAg (N = 34) **		
**Age (years)**				
< 25	158(33.3)	9(5.7)	1	
25-29	203(42.7)	13(6.4)	1.13(0.47-2.72)	0.78
≥30	114(24.0)	12(10.5)	1.95(0.79-4.79)	0.15
**Residence **				
Urban	323(68.0)	25(7.7)	1.33(0.61-2.93)	0.47
Rural	152(32.0)	9(5.9)	1	
**Education**				
Illiterate	59(12.4)	4(6.8)	1.02(0.27-2.77)	0.98
Elementary	165(34.7)	9(5.4)	0.81(0.28-2.35)	0.69
Secondary	161(33.9)	15(9.0)	1.44(0.54-3.85)	0.47
College and above	90(18.9)	6(6.9)	1	
**Occupation **				
Housewife	246(51.8)	20(8.1)	2.74(0.36-21.17)	0.33
Employed	133(28.0)	12(9.0)	3.07(0.39-24.56)	0.29
Daily labor	12(2.5)	1(8.3)	2.82(0.16-49.00)	0.48
Merchant	52(10.9)	0	0.00	0.99
Student	32(6.7)	1(3.1)	1	
**Marital status **				
Married	428(90.1)	33(7.7)	1.42(0.18-11.01)	0.74
Single	29(6.1)	0	0.00	0.99
Divorced /Widowed	18(3.8)	1(5.6)	1	
**Gestational age **				
1^st^ trimester	95(20.0)	9(9.5)	1.88(0.72-4.92)	0.19
2^nd^ trimester	171(36.0)	9(5.3)	1	
3^rd^ trimester	209(44.0)	16(7.7)	1.49(0.64-3.47)	0.35
**Gravidity**				
Primigravida (1)	153(32.2)	10(6.5)	1	
Multigravida (≥2)	322(67.8)	24(7.5)	1.15(0.54-2.47)	0.72

*∗*Data were shown as N (%); N: number; HBsAg: hepatitis B surface antigen.

**Table 2:** Risk factors associated with HBsAg positivity among pregnant women in Yirgalem Hospital, October 2015 to August 2016.

Risk factors	Total women	HBsAg (N=34)	COR(95%CI)	aOR(95%CI)	p-value
	*∗*(N = 475)	*∗*Positive (%)			
Place of birth					
No birth	146(30.7)	8(5.5)	1		
Home	93(19.6)	7(7.5)	1.40(0.49-4.01)		
Health institution	236(49.7)	19(8.1)	1.51(0.64-3.55)		
Surgery					
Yes	82(17.3)	7(8.5)	1.27(0.53-3.01)		
No	393(82.7)	27(6.9)	1		
Blood transfusion					
Yes	23(4.8)	1(4.3)	0.58(0.08-4.42)		
No	452(95.2)	33(7.3)	1		
Tattooing					
Yes	91(19.2)	5(5.5)	0.71(0.27-189)		
No	384(80.8)	29(7.6)	1		
Tooth extraction					
Yes	132(27.8)	8(6.1)	0.79(0.35-1.78)		
No	343(72.2)	26(7.6)	1		
Multiple sexual partner					
Yes	43(9.1)	7(16.3)	2.92(1.19-7.16)	2.94(1.17-7.41)	0.02
No	432(90.9)	27(6.2)	1		
Family history of liver disease					
Yes	23(4.8)	1(4.3)	0.58(0.08-4.42)		
No	452(95.2)	33(7.3)	1		
Abortion					
Yes	67(14.1)	5(7.5)	1.05(0.39-2.82)		
No	408(85.9)	29(7.1)	1		
DM					
Yes	28(5.9)	3(10.7)	1.61(0.46-5.63)		
No	447(94.1)	31(6.9)	1		
HIV					
Yes	48(10.1)	10(20.8)	4.42(1.97-9.93)	4.44(1.96-10.08)	0.001
No	427(89.9)	24(5.6)	1		

*∗*Data were shown as N (%); N: number; HBsAg: hepatitis B surface antigen; DM: diabetes mellitus; HIV: human immunodeficiency virus.

**Table 3:** Relation of HBsAg and previous status of participants to HBV, vaccination, andawareness of pregnant women about route of transmission in Yirgalem Hospital, October 2015 to August 2016.

Participant responses	Total women	HBsAg
	(N = 475)	Positive(N=34)
Previously screened for HBV	74(15.6)	6(8.1)
Previous HBV result, positive	3/74(4.1)	3(100)
Have you taken HBV vaccine? Yes	12(2.5)	0
Is HBV transmitted sexually? Yes	221(46.5)	15(6.8)
Is HBV transmitted through contact of blood and body fluid? Yes	213(44.8)	15(7.0)
Is HBV transmitted from mother to child? Yes	168(35.4)	12(7.1)
Other routes of transmission mentioned	154(32.4%)	11(7.1)

HBsAg: hepatitis B surface antigen; HBV: hepatitis B virus.
